# About 4-day rhythm of proliferative activity of fibroblast-like cell cultures isn’t endogenous and don’t depend from the variations of Earth’s magnetic field

**DOI:** 10.1038/s41598-022-11191-y

**Published:** 2022-05-03

**Authors:** Marina A. Diatroptova, Anna M. Kosyreva, Mikhail E. Diatroptov

**Affiliations:** 1 A.P. Avtsyn Research Institute of Human Morphology, Petrovsky National Research Centre of Surgery, Tsyurupi str 3, Moscow, 117418 Russia; 2 Medical Institute of Peoples’ Friendship University of Russia (RUDN University), Moscow, Russia; 3 A.N. Severtsov Institute of Ecology and Evolution, Russian Academy of Science, Moscow, Russia

**Keywords:** Cell growth, Mitosis, Circadian rhythms

## Abstract

A study of the 4-day rhythm of the proliferative activity of the embryonic fibroblast-like cells in the logarithmic growth phase was carried out. It was shown that in cell cultures obtained on different days from embryos of different ages, the phase of the 4-day rhythm coincides. In vitro the maxima of the proliferative activity were consistent with the minima of the motor activity of mice. Freezing the culture for 2 or 6 days does not cause a shift in the phase of the 4-day rhythm of cell proliferative activity compare with the unfreezing culture. That indicates the existence of an external synchronizer, which determines the 4-day infradian rhythm of the proliferative activity of embryonic cells. Then we daily thawed samples of single L929 culture of mice fibroblast-like cells for 22 and 17 days and researched the dynamics of its proliferative activity. We also showed 4-day rhythm of the simultaneous increase in the number of cells for all thawed samples. Taking into account that deep freezing of a culture leads to the cessation of all life processes, the fact we obtained indicates an exogenous mechanism of the formation of about a 4-day rhythm of the proliferative activity of cell culture. Variations of the Earth's magnetic field could be one of the external synchronizers of the infradian rhythm. We studied the increase in number of L929 cell in conditions of a magnetic permalloy screen and showed that the magnetic shielding no affect the parameters of the infradian rhythm of L929 cell proliferative activity. So further searches of the external synchronizers are need.

## Introduction

Infradian about 4-day fluctuations were found in many biological parameters: concentrations of melatonin, glucocorticoid and sex hormones^1–6^, motor activity^7^, immune system^8,9^, mitotic activity of cells^10,11^. Based on the facts that (1) 4-day rhythms are preserved during the removal both of the testes and adrenal glands^12^, and (2) hibernating hedgehogs with reduced hormonal activity had the stable rhythms of spontaneous short-term arousals^13^, it can be argued that the formation of this infradian rhythm is not associated with hormonal regulation. It is interesting that the removal of the pineal gland, which is involved in the formation of circadian rhythms^14^, also does not affect the period and phase of the 4-day rhythm of mitotic activity of the rat esophagus epithelium^7^. Probably, the 4-day rhythm is determined by the activation of the sympathetic nervous system, which is indicated by the presence of this rhythm in the dynamics of epinephrine excretion in the urine^2^, as well as the whole complex of signs associated with an increase in motor activity, the release of glucocorticoid hormones, suppression of immune reactions, a decrease in proliferative cell activity.

The mitotic activity of epithelial cells is the convenient model of the stable manifestation of 4-day rhythm^11^. However, the formation mechanisms of this rhythm have not been established yet. In vivo studies do not allow to exclude the influence on the mitotic activity of cells of humoral factors of the body internal environment, that have a 4-day rhythm determined by the activation of the sympathetic nervous system. Based on this hypothesis, the 4-day rhythm of mitotic activity in an isolated cell culture should be absent. However, studying the daily dynamics of the proliferative activity of the culture of embryonic fibroblast-like cells in the logarithmic growth phase, we revealed a 4-day rhythm that persists for at least three weeks of cultivation^15^.

There are three possible variants of this rhythm’s origin: (1) completely endogenous, self-sustaining on the basis of molecular genetic interactions, and, therefore, such a rhythm will not be synchronous in different cell cultures; (2) completely exogenous, caused by the influence of an external environmental factor; (3) endogenous, but having an external synchronizer, and, therefore, these two rhythms will be synchronous.

To determine the possible origin of the 4-day rhythm of the proliferative activity of fibroblast-like cells, it is necessary to establish:

1. are the manifestations of the 4-day rhythm of proliferative activity in cultures obtained on different days from embryos of the same age synchronous?
2. does the phase of the rhythm in cell culture comparable with that in vivo? Under in vivo conditions, the phase of the infradian rhythm of the motor activity of animals is observed in antiphase to the 4-day rhythm of mitotic activity (mitotic index) of the esophageal epithelium^7^.
3. will freezing of cells for a period not multiple of 4 days cause a phase shift of the investigated infradian rhythm relative to the intact culture?

However, immediately after thawing, embryonic fibroblast-like cells do not begin to divide intensively. It is a considerable time period between the isolation of the culture and the beginning of the experiment, during which the cultures can synchronize their endogenous rhythms. Therefore, at the final stage of our research, we studied the changes in the proliferative activity of the fiboroblast-like cells mice culture L929, which has a high proliferative potential. According to our latest data a proliferative activity of L929 cells have the about 4-day infradian rhythm too^16^. The aim of the 4th point of the study was: to establish the exogenous/endogenous nature of about 4 days of the rhythm of proliferative activity of fibroblast-like L929 cells by daily thawing of same frozen samples of the same culture. If, after defrosting, the rhythm is immediately observed in the phase, as if it were not stopped, then we can suggest its exogenous origin, but if the restoration of the rhythm phase is observed gradually, then this is an endogenous rhythm that has an external synchronizer.

Variations of the Earth's magnetic field could be one of the external synchronizers. In order to assess the contribution of these fluctuations to the formation of the about 4-day rhythm of cell proliferative activity, we decided:

4. to study the rhythmicity of the proliferative activity of the L929 mice cells in conditions of the electromagnetic shielding.

## Results

### Infradian rhythm of increase of embryonic cells’ number

**Figure 1:**
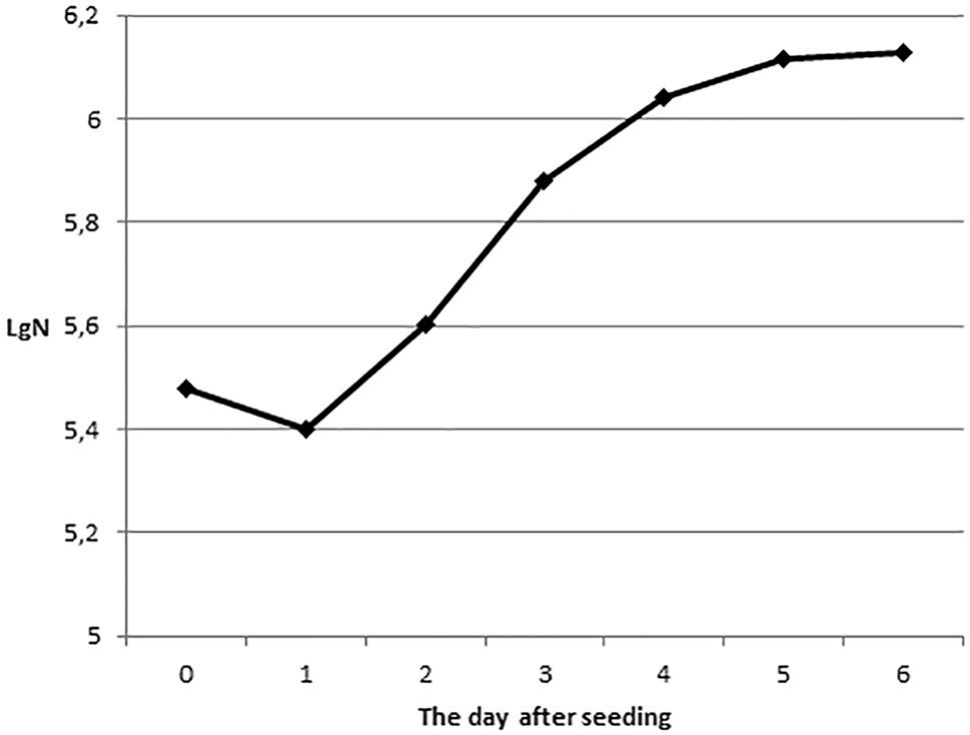
Typical growth curve of the cell culture of embryonic fibroblast-like cells.

The highest rate of the number of embryonic fibroblast-like cells increase was observed on the second and third days after sowing, when the cultures were in the phase of logarithmic growth. Following gradual decrease in growth rates was recorded due to contact inhibition of cells (Fig. 1). As an indicator of proliferative activity, the daily increase in the number of cells on the second and third days of cultivation was calculated.

In order to establish the synchronicity of the 4-day rhythm, the proliferative activity of fibroblast-like cells obtained from embryos conceived on different dates—November 4 and 6, 2019, and November 17 and 19, 2019 was investigated. The difference in the date of conception of the studied embryos was 2 days, which corresponds to half period of the 4-day rhythm.

**Figure 2:**
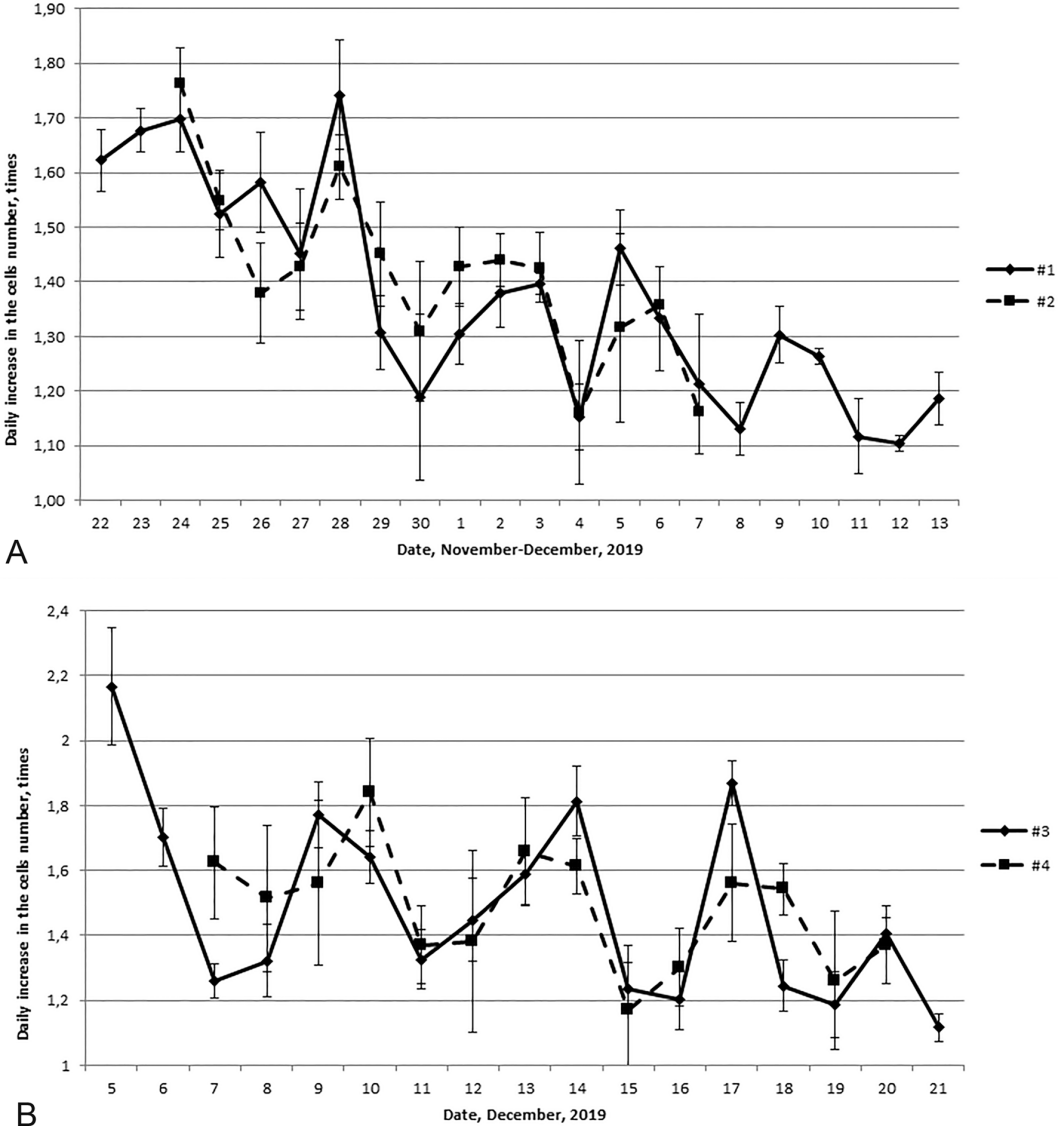
Dynamics of the daily increase in fibroblast-like cells number during the period of the logarithmic growth state obtained in different phases of the 4-day rhythm: (**A**) row 1—November 16, 2019; row 2—November 18, 2019. (**B**) Row 1—November 29, 2019; row 2—December 1, 2019.

Figure 2A shows daily cell growth rates in two cultures, one of which was isolated on November 16 from embryos conceived on November 4, and the other—on November 18 from embryos conceived on November 6. The correlation coefficient between the indicators of these cultures was r = 0.67 (p = 0.008), therefore, their dynamics can be considered synchronous. The maximum indicators of the increase in the cells number in both cultures were noted on November 24, 28, December 2–3, 5–6 and 9–10. The minimum indicators of the increase in the cells number were registered on November 26 and 30, December 4, 8 and 12. So that we observed about 4-day rhythm.

In the first 12 days, the growth of the culture was high. We found out a statistical difference of the daily cells number increase between the acrophase (November 24, 28 and December 2) and bathyphase (November 26, 30 and December 4) of the 4-day rhythm. At the acrophase, the cells number increased 140 (120; 220) thousand cells and at the bathyphase, the increase was only up on 60 (40; 120) thousand cells (p = 0.006). The existence of a 4-day rhythm was confirmed by autocorrelation analysis. As the cell growth rate gradually decreased, its deviations from the approximating trend line were calculated. The autocorrelation coefficients are presented in the Table 1. The correlation coefficient between the original series and the series shifted by 2 days is r = − 0.36 (p = 0.11), and by 4 days—r = 0.49 (p = 0.04).

**Table 1:** The calculated autocorrelation coefficients for the dynamics of the mean of proliferative activity of the culture cells #1 and #2, received on November, 16 and 18, 2019.

lag, day	r	p
1	− 0.07	0.73
2	− 0.36	0.11
3	0.002	0.99
4	0.49	0.04

Figure 2B shows the daily growth of two other cells of cultures. The first culture was isolated on November 29 from embryos who had been conceived on November 17. 2019. The second one was isolated on December 1 from embryos who had been conceived on November 19. We also revealed the synchronicity in the dynamics of these cultures and coefficient of correlation (r) was 0.68 (p = 0.007). The maximum indicators were registered on 5–6 and 9–10, 14 and 18 December, and the minimum—on 8, 12, 16 and 19–20 December. The indices of daily growth in the acrophase of the 4-day rhythm were 160 (130; 280) thousand cells, and in the bathyphase were 100 (70; 135) thousand cells. The daily growths in the acrophases and the bathyphases statistically significantly differed among themselves (p = 0.01). The autocorrelation coefficient between the original series and the series shifted by 2 days is statistically significant and the coefficient correlation (r) is − 0.69 (p = 0.002), which also confirms the stability of the 4-day rhythm in the dynamics of proliferative activity of culture cells.

In all four studied cultures, the maximum proliferative activity got in the same calendar dates; therefore, the phase of the 4-day rhythm of these cultures coincided.

### Matching of infradian rhythms between proliferative activity of embryonic cell culture and motor activity of mice

**Figure 3:**
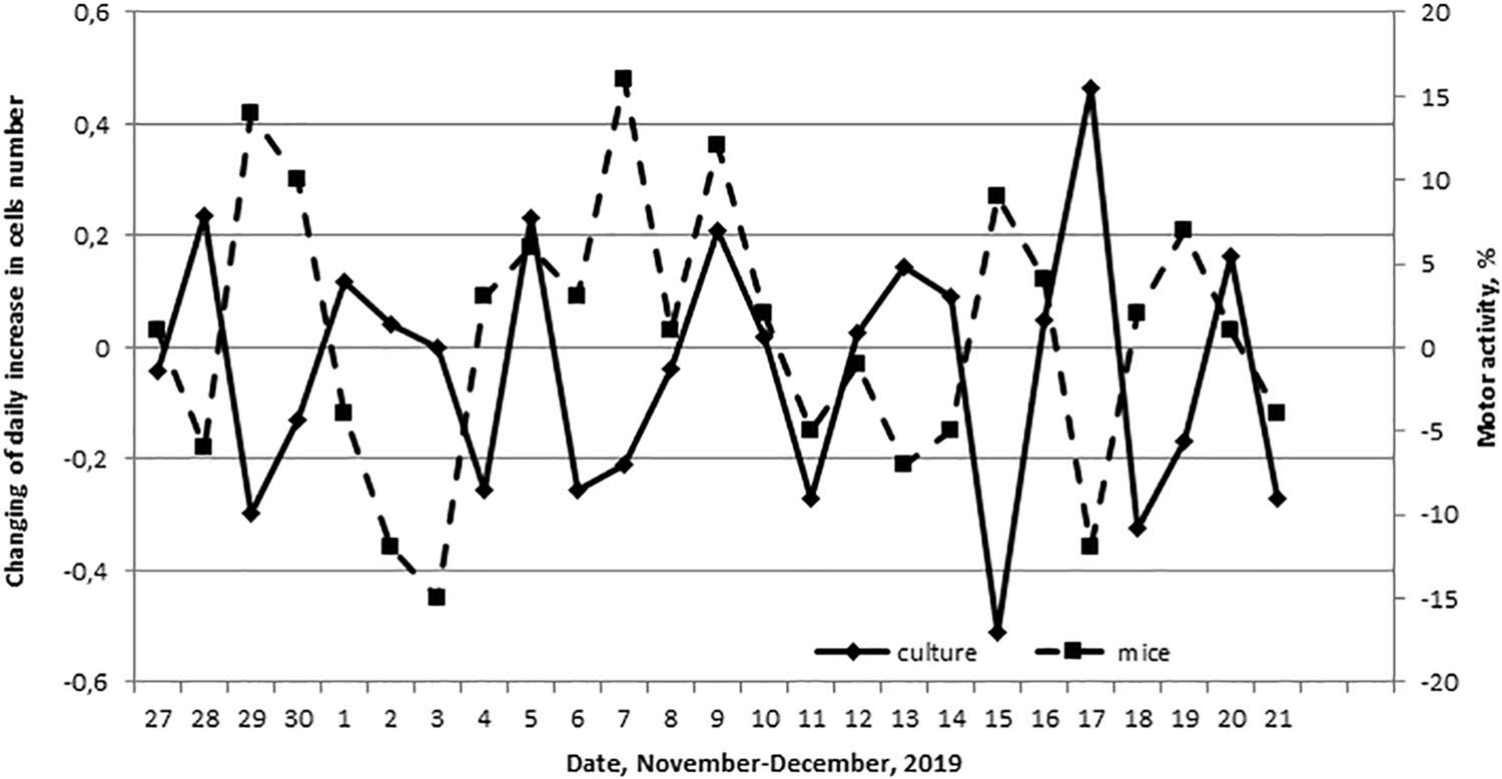
Dynamics of the deviation of the daily increase in the fibroblast-like cells number from the trend line (linear approximation) in comparison with the total motor activity of male mice (n = 7); the percentage deviations from the individual mean level are presented.

We investigated the daily motor activity of male mice in the period from November 27 to December 21, 2019 to identify the synchronicity of the 4-day rhythm of proliferative activity in vitro and the motor activity of mice in vivo (Fig. 3). The maxima of the motor activity of mice were at the minima of the proliferative activity of the culture cells. A negative correlation coefficient was revealed between these parameters: r = − 0.47 (p = 0.016).

### Freezing and thawing of embryonic cell cultures

**Figure 4:**
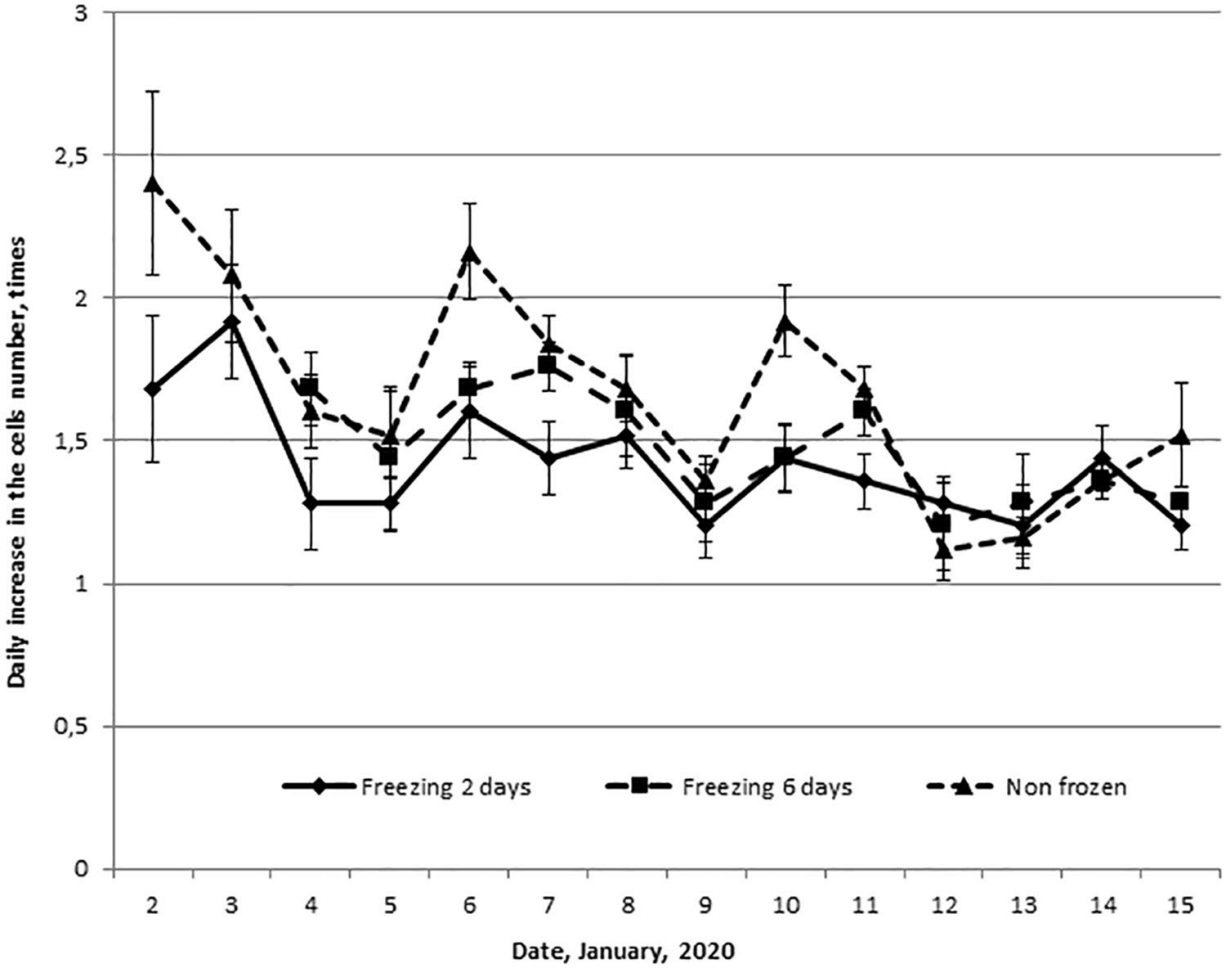
Dynamics of the daily increase in the fibroblast-like cells number of single culture, some of them were frozen for 2 and 6 days (rows 1 and 2), and some were cultured as usual (row 3).

To test the hypothesis about the endogeneity of the 4-day rhythm, we froze the cells for a period not multiple of 4 days. In the case of the endogenous nature of this rhythm, such a procedure should have led to a shift the phase of the proliferative activity of the frozen cell culture in relation to the intact one. Figure 4 shows the daily increase in the cells number of one culture, a part of that were frozen for 2 and 6 days, and the other part were cultured as usual. We detected a 4-day rhythm of the dynamics of proliferative activity of all three cultures. The maximum indicators were noted on January 2–3, 6–7 and 10–11, and the minimum on January 4–5, 8–9 and 12–13. The correlation coefficient between frozen and intact cultures was r = 0.78 (p < 0.001) and r = 0.72 (p = 0.008). Consequently, freezing the culture for 2 and 6 days did not lead to a change in the phase of the 4-day rhythm of proliferative activity relative to the intact one.

### Freezing and thawing of L929 cells

**Figure 5:**
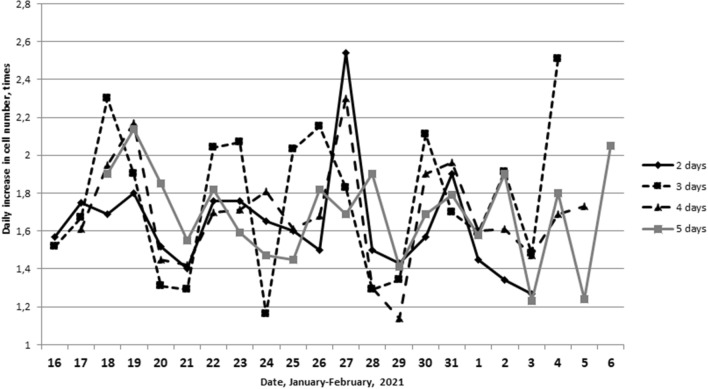
Dynamics of the daily increase in the L929 cells number in the samples of the culture, analyzed in 2, 3, 4 and 5 days after thawing.

The next task was to study the dynamics of proliferative activity of single L929 cell culture samples, which were thawed daily and immediately plated into Petri dishes (Table 4, first series). Figure 5 shows the dynamics of the daily increase in the cell number of the studied cultures after the 2nd, 3rd, 4th and 5th days of cultivation. It can be noted that synchronous changes are observed in the dynamics of proliferative activity. Indicators of the daily increase in the number of cells have maximums on January 18–19, 22–23, 27, 30–31 and February 4. The minimum increase in the cell number was noted on January 16, 21–21, 24, 28–29, February 1 and 5, as well as an additional minimum on February, 3. The correlation coefficient of the increase in the cell number is maximum between 2 and 3 days (r = 0.54, p = 0.024).

**Figure 6:**
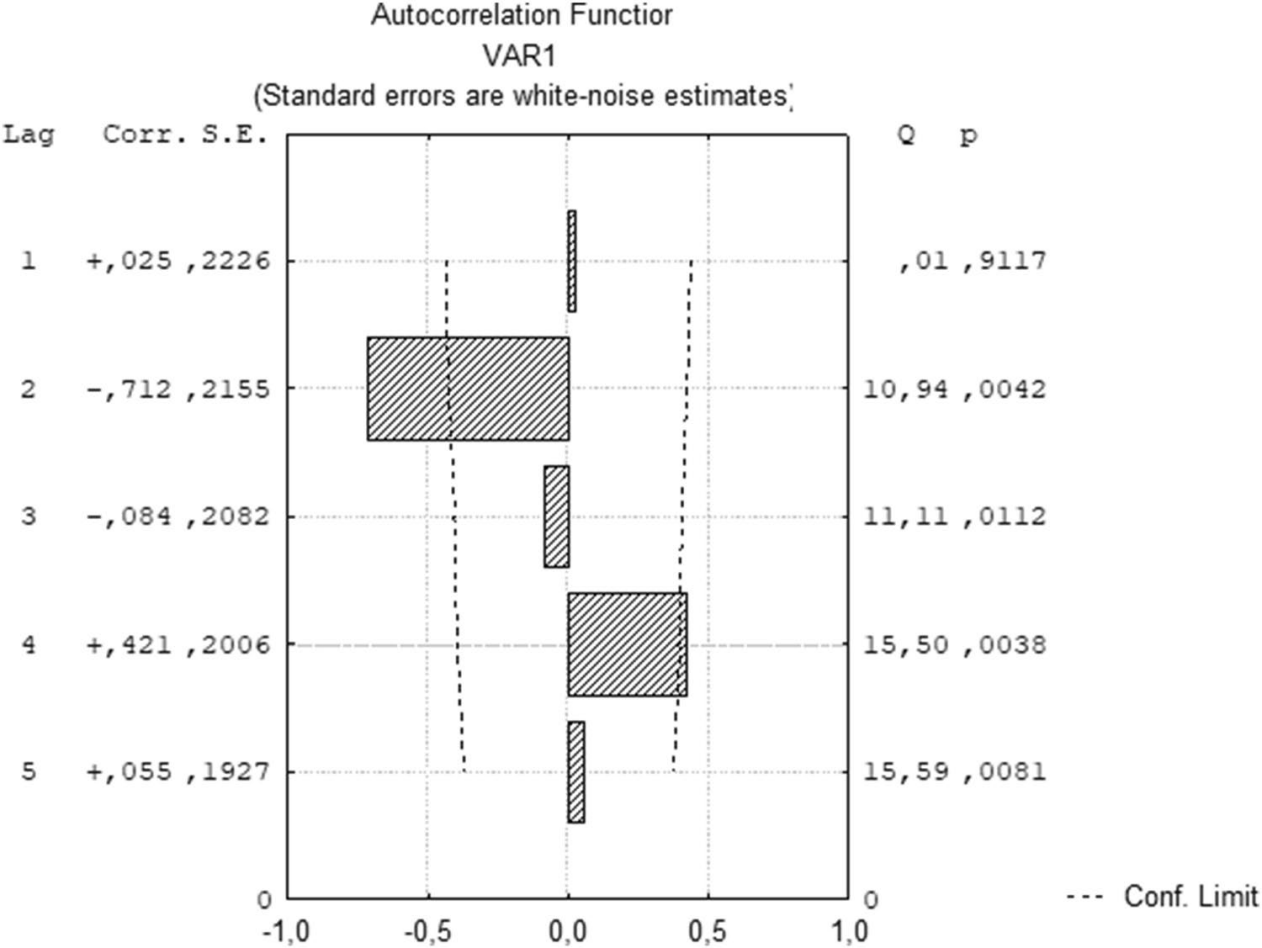
Autocorrelation function of the dynamics of the daily increase in the number of L929 cells from January 16, 2021 to February 1, 2021.

To approve the discovered rhythm of L929 cell proliferative activity for the period from January 16 to February 1, the autocorrelation coefficient of the average index of proliferative activity for the 2nd–5th days of cultivation was calculated (Fig. 6). Autocorrelation analysis confirms the statistical significance of the 4-day rhythm.

Thus, in the dynamics of the L929 cell proliferative activity over a long period from January 16 to February 1, a 4-day rhythm was revealed. During the acrophase the indicator of daily growth after the 2nd and 3rd days of cultivation (January, 18–19, 22–23, 27, 30–31) was 1.97 (1.69–2.12) and during the bathiphase (January, 16, 20–21, 24, 28–29) was equal to 1.40 (1.30–1.61). There was a statistically significant difference between the values in acrophase and bathiphase (p = 0.0015).

Based on the fact that the maximum correlation coefficient is observed between the 3 and 4 days of an increase in the L929 cells number, it can be assumed that it is during this period that the culture is most sensitive to external influences that affect the proliferative activity of cells. The indicators of 2 days after the start of cultivation have great instability, probably caused by the procedure of thawing and sowing the cells. The indicator of proliferative activity after 5 days of cultivation has a small value, which is probably affected by the exhaustion of the nutrient medium and the high density of the monolayer formed by this time. Therefore, in the next series of experiments, we analyzed the proliferative activity after 3 days of cultivation.

**Figure 7:**
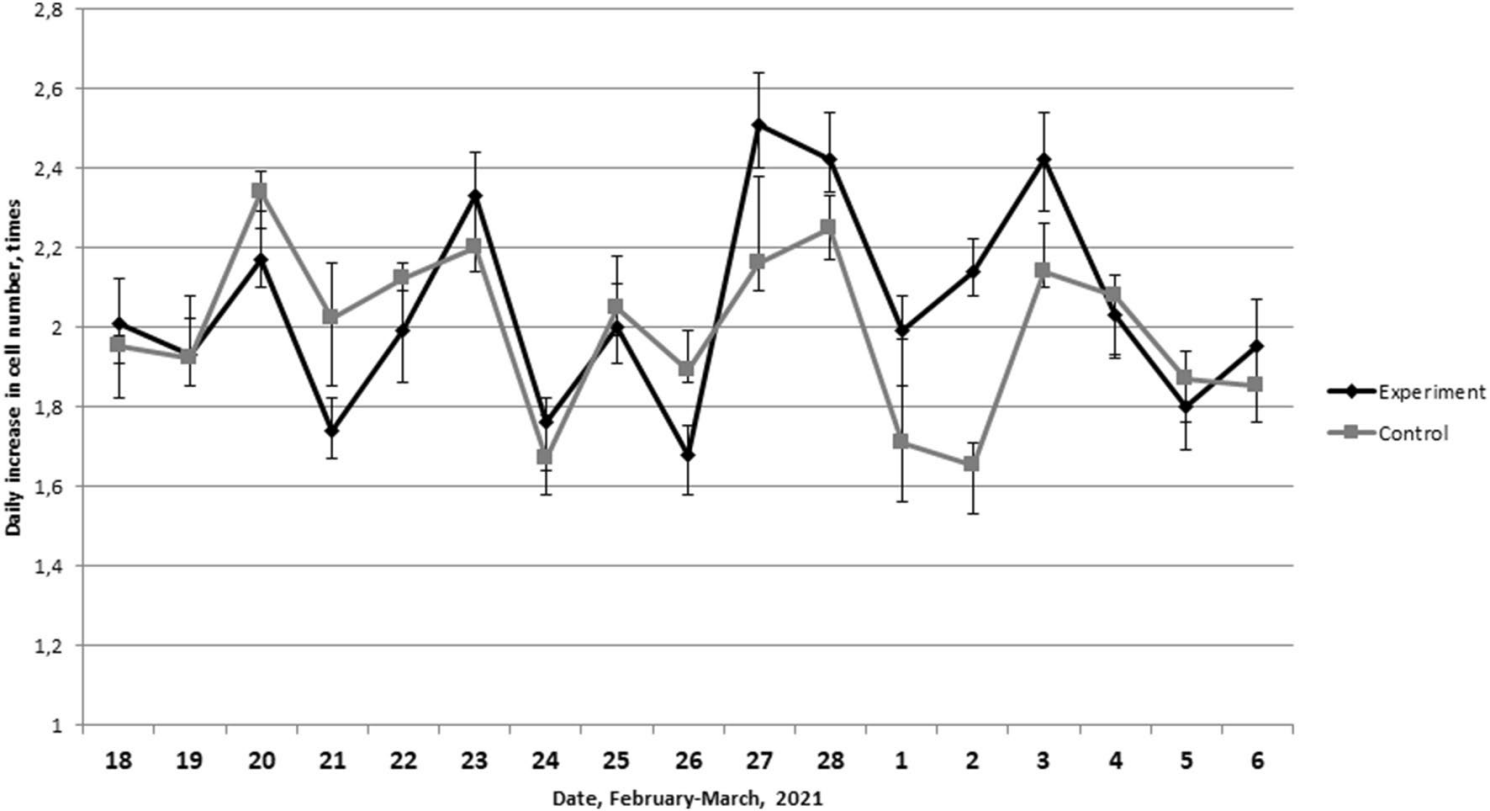
Dynamics of the daily increase in the L929 cells number after thawing (experiment) and in not frozen culture (control).

In the second series of experiments on daily thawing of cryoprobes in the period February 15–March 3, 2021 (Table 5), a similar dynamic was revealed. The maximum daily increases in the cell number were observed on February 20, 23, 27 and March 3 (Fig. 7). The minimum values of proliferative activity were established on February 21, 24–26, March 1 and 5. It should be noted that a statistically significant correlation is observed between experimental samples, that were daily thawing and control samples that were parallel cultivated and not subjected to freezing (r = 0.65, p = 0.004).

### The rhythmicity of the proliferative activity of the L929 mice cells in conditions of the electromagnetic shielding

To assess the contribution of the variability of the Earth's magnetic field to the 4-day infradian biological rhythm of the L929 cell proliferative activity, we studied the increase in the number of cells in a L929 culture located in a magnetic permalloy screen that 35-fold attenuates the total geomagnetic field. Two aliquots obtained from one culture were thawed daily, one of them was sown on Petri dishes and placed in the magnetic screen, and the other was sown on Petri dishes and incubated without a magnetic screen.

**Figure 8:**
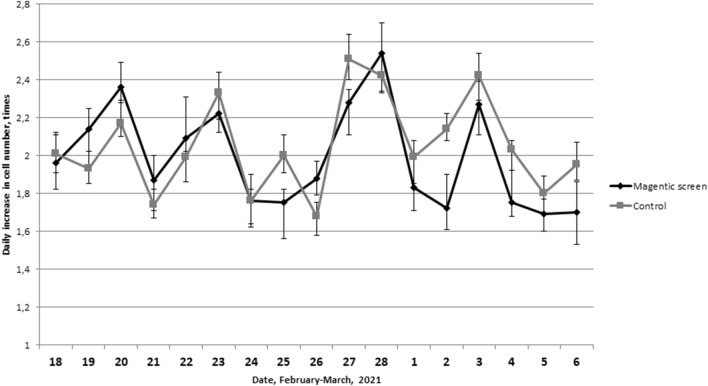
Dynamics of the daily increase in the number of L929 cells in the condition of the permalloy magnetic screen and without it. There are data obtained 3 days after the begin of cultivation in the graph.

The dynamics of the daily increase in the number of L929 cells in the permalloy magnetic screen and without it was similar (Fig. 8). The correlation coefficient between the parameters in the experimental and control groups was r = 0.56 (p = 0.019). The magnitude of the range (doubled amplitude) between the readings of the daily increase in the number of cells at the points of minimum and maximum for the culture placed in the magnetic screen was 0.48 times (0.45; 0.53) times, and for the culture outside the magnetic screen 0.47 times (0.43; 0.52), which is not has a statistically significant difference.

Thus, shielding from the Earth's magnetic field does not affect the parameters of the infradian rhythm of proliferation.

## Discussion

In cell cultures obtained from embryos of the same age, but conceived on different calendar dates, the phase of the 4-day rhythm of proliferative activity coincides. Therefore, there is an external effect that synchronizes the proliferative activity of fibroblast-like cells in vitro. A similar pattern was previously established by us for in vivo conditions: the 4-day rhythm of glucocorticoid hormones and the mitotic activity of epithelial cells of the esophagus coincides in phase not only between individuals of the same species, but also between species belonging to the class of mammals and birds^17^. Consequently, the phase of the 4-day rhythm of the proliferative activity of the primary cell culture in vitro continues to be synchronized with the analogous rhythm of mitotic activity in vivo.

Freezing the culture for 2 or 6 days (a period not multiple of 4 days) does not cause a shift in the phase of the 4-day rhythm of the proliferative activity of embryonic fibroblast-like cells relative to the intact culture. Thus, the studied rhythm is, most likely, either a consequence of the direct action of an external factor on the cells, i.e. exogenous, or endogenous, but having an external synchronizer as after thawing the culture, before the start of the experiment, it was necessary to carry out one passage for 5 days, perhaps, the cells managed to synchronize their endogenous rhythms with an external factor during this short period.

A long-term study of the L929 cell culture proliferative activity during to simultaneously freezing of single samples and then thawing daily for 20 days, showed existence of a 4-day rhythm. We suggest that as after deep freezing all vital processes stop in the cells, the presence of a 4-day rhythm in the daily increase in L929 cell number is caused an exogenous mechanism of the formation of this rhythm. The hypothesis that the studied rhythm is endogenous, but it is synchronized by an external factor, isn’t consistent, as we observed the rhythm synchronicity already two days after defrosting. If the synchronization by a not constantly acting external environmental factor was being, synchronous rhythmic changes in proliferative activity would be observed later after defrosting.

We suggest that the around 4-day rhythm of proliferative activity has a universal character.

In vivo, such rhythms were found both in rat esophageal epithelial cells in mice and quails^11^, belonging to different classes of animals. Focan et al.^10^ showed a similar infradian rhythm of proliferative activity in mouse sarcoma cells. Since 4-day rhythms of cell proliferative activity are observed in different classes of animals and on different types of cells, it can be assumed that either these rhythms are endogenous and have an external heliogeophysical synchronizer, or they are completely exogenous. The biological infradian rhythms in vitro on other cell cultures (not fibroblast-like cells) have not been studied yet. In this article, the non-endogenous nature of this infradian biological rhythm was demonstrated. That suggests the mitotic activity of cells of all types is under the regulatory influence on an external environmental factor which have not been unidentified yet. It is logical to assume that different types of cells have different sensitivity to this environmental factor and, consequently, different degrees of manifestation of the 4-day rhythm of proliferative activity.

Specific markers of cell proliferation are Ki-67 and cyclins. Ki-67 protein is present during all active phases of the cell cycle (G1, S, G2, and mitosis), but is absent from G0 phase^18^. Therefore, for cell cultures with a high degree of proliferation, including embryonic fibroblast-like cells and L929 cell culture, this marker is insignificant, since Ki-67 is expressed in more than 90% of these cell. To identify the most sensitive to an external synchronizer stage of the cell cycle, it is advisable to use other markers, such as cyclins^19,20^.

Another approach to establishing the molecular mechanism of the formation of infradian rhythms is the search for tumor cell cultures that do not have infradian rhythms of proliferation. Knowledge of specific molecular disturbances for such cultures, leaded to the loss of inhibition of proliferation, will allow to appeared molecular biological pathways for the regulation of infradian rhythms of the cell cycle.

Infradian rhythms are observed not only in the proliferative activity of cells. Previously, we have shown that ex vivo production of pro-inflammatory and anti-inflammatory cytokines by spleen cells has a 4-day rhythm^21^. Infradian rhythms of cytokine concentration were detected in the blood of patients with melanoma^9^. There were more stable biorhythms of a larger number of immune parameters [such as IL-12p70, IL-1RA, IL-9, IL-10, IL-13, IL-15, IL-17, G-CSF, VEGF, Th1 cells (CD4^+^TIM3^+^), Th2 cells (CD4^+^CD294^+^), Tregs (CD4^+^CD25^+^FoxP3^+^), type 1 dendritic cells (CD11c^+^HLA-DR^+^), type 2 dendritic cells (CD123^+^HLA-DR^+^), type 1 macrophages (CD14^+^CD197^+^) and type 2 macrophages (CD14^+^CD206^+^)] in patients with metastatic melanoma^9^. However, in vitro the infradian rhythms of the cell functional activity have not been studied yet. In turn, ultradian clock rhythms are found in almost all types of cellular activity: protein synthesis, oxygen consumption, enzyme activity, ATP concentration, pH, changes in cell mass. These rhythms are characteristic not only for unicellular organisms, but also for mammalian cell cultures^22,23^.

Thus, our results point out an exogenous mechanism of a 4-day rhythm formation of the daily increase in cells number both in embryonic and L929 cultures. The direction for further research is the search for an external environmental factor that determines the rhythmic changes in the proliferative activity of cell culture.

Oscillations of the geomagnetic field can be a possible external synchronizer. It was found that *the 30-fold* screening of the Earth's magnetic field causes a decrease in the proliferative activity of the Fibrosarcoma HT1080 tumor cells and colorectal HCT116 cancer cells^24^. In^25^ it was shown that 24-h exposure of the endothelial cell culture in a weak constant magnetic field leads to an increase in their proliferation.

Currently, there are reasons to consider natural electromagnetic fields as a possible synchronizer of infradian and ultradian rhythms^26,27^. In addition to the established mechanism for the perception of these oscillations at the whole organism^28–30^, there are few data that the reception of electromagnetic oscillations is possible within the one cell. It was shown that irradiation of a suspension of T- and B-lymphocytes with low-intensity electromagnetic radiation (42.2 GHz, 1 μW/cm^2^ amplitude modulation 10 Hz) suppressed their blast transformation^31^. Effecting of periodic weak outside pulsed magnetic fields to the activated neutrophils, attached to a glass substrate, increase by them the production of reactive oxygen species and NO^32^. To manifest this effect, the frequency of the external magnetic effect must coincide with the natural frequency of concentration fluctuations NAD(P)H in a cell, which is about 20 s, and the presence of calcium ions in the medium is necessary for the perception of an external magnetic (or electric) field, which indicates the participation of calcium channels in magneto reception^32^.

In order to test this assumption, a permalloy magnetic screen was used that 35-fold attenuates the total geomagnetic field. The dynamics of the daily increase in the number of cells with and without magnetic shielding had a high positive correlation. The amplitude of about 4-day fluctuations in the proliferative activity of the culture located in the magnetic screen did not decrease compare with the control group. Consequently, either the mechanism of formation of this infradian rhythm is not depend on to the Earth's magnetic field, or the cells have an extremely highly sensitive system of reception of geomagnetic field variations, which allows not changing the value of the response biological reaction with a 35-fold decrease in the value of the geomagnetic signal.

Previously, we established a relationship between the dynamics of motor activity of mice and the intensity of fluctuations of secondary cosmic rays near the Earth's surface^33^. The daily increases in the number of fibroblast cells in vitro had a negative correlation with the intensity of fluctuations of the secondary cosmic radiation^34^. Despite the correlation between cell proliferative activity and cosmic radiation fluctuations, this heliogeophysical factor can hardly be considered as an external synchronizer of 4-day infradian rhythms. Because at first, there is no correlation between the cell proliferative activity and the absolute value of the flux of secondary cosmic rays, which, in turn, can affect the natural radiation background. Secondly, the value of fluctuations of counting rate of the secondary cosmic radiation is about 0.1% of their total flux, and the secondary cosmic radiation flux itself has also low intensity: about 1 event per minute per 1 cm^2^. In other words, 0.1% fluctuations of a low-intensity flux of secondary cosmic rays cannot even theoretically determine 20–30% of changes in the proliferative activity of fibroblast-like cells.

It should be noted that the established ultradian rhythms of motor activity and body temperature in mammals and birds mainly coincide with the periods of natural oscillations of the Earth^35^. In turn, the spectrum characteristic of the Earth's own oscillations is observed in the dynamics of various geophysical factors—microseismic activity, fluctuations of the geomagnetic field, microfluctuations of atmospheric pressure, fluctuations of the counting intensity of secondary cosmic rays, and even radioactive decay^35,36^. Further searches for an external factor that determines the ultradian and infradian rhythms of motor activity of animals and the cell proliferative activity should be carried out in the field of atmospheric physics, the dynamics of its internal gravitational waves and atmospheric pressure microfluctuations. Revealing the nature and parameters of an external factor regulating proliferative activity of isolated cells will make it possible to develop a method for modulating regeneration processes.

Based on the exogenous mechanism of the about 4-day rhythm formation of the cell culture proliferative activity and indirect evidence of the coincidence of the phase of the 4-day rhythm of the proliferative activity of the cell culture in vitro with the similar rhythm of mitotic activity in vivo, it can be suggested that the infradian rhythms of other biological parameters in vivo are caused by a constantly or almost constantly manifested rhythmic factor of the environment. However, this statement requires further experimental verification.

## Material and methods

### Animals

3 male and 10 female C57Bl/6 mice were used for obtained embryonic fibroblast-like cells. 7 male C57Bl/6 mice were used for estimation of motor activity. All animals were purchased from the “Stolbovaya” branch of the Federal State Budgetary Institution of Science and Technology of the Federal Medical and Biological Agency of Russia. The study received permission from the Bioethics Committee of the Research Institute of Human Morphology (Protocol No. 24, September 2, 2020). The study is done in accordance with ARRIVE guidelines (https://arriveguidelines.org) and all methods were carried out in accordance with relevant guidelines and regulations. All manipulations with animals were carried out according to the European convention for the protection of vertebrate animals used for experimental and other scientific purposes (ets no. 123), Strasbourg, 2006, and all efforts were made to minimize the suffering and distress of animals. 5 female mice per cages (40 × 14.5 × 24 cm) were housed in temperature-regulated room at 12:12 h light–dark cycle, relative humidity, between 55 and 65%; and unlimited access to water and food (“Char”, JSC “Range-Agro”, Russia). Male mice (n = 10) were kept in individual plastic cages (40 × 14.5 × 24 cm).

### Obtaining of embryonic fibroblast-like cells

Fibroblast-like cells were isolated from 12–13th day embryos of pregnant C57Bl/6 female mice. In this period of the development an embryo contains a high percentage of undifferentiated mesenchyme, which is the main source of fibroblasts^37^. In order to establish a dated gestation date, female mice were housed with males for only one night. Accordingly, after 12 days, the females were scarified by dislocation of the cervical vertebrae. For the following study embryos were taken without internal organs and head. In total, 5 cultures were obtained in the work from 5 pregnant female mice respectively (Table 2). To obtain a culture, embryos were taken from one female. The fibroblast-like cells obtained from different embryos of one female were mixed with each other. The number of embryos taken depended on the volume of cell culture required for the experiment.

**Table 2:** Calendar date of experimental manipulations with embryonic cells culture from November, 2019 to January, 2020.

Number of culture	Conception date	Date of separation of the primary culture	Freezing date	Defrost date	Initiating date of culture daily sowing on Petri dishes	Initiating date of daily cell counting	Finished date
1	Nov, 4	Nov, 16	–	–	Nov, 20	Nov, 21	Dec, 13
2	Nov, 6	Nov, 18	–	–	Nov, 22	Nov, 23	Dec, 7
3	Nov, 17	Nov, 29	–	–	Dec, 3	Dec, 4	Dec, 21
4	Nov, 19	Dec, 1	–	–	Dec, 5	Dec, 6	Dec, 20
5	Dec, 3	Dec, 16	Dec, 20	Dec, 26	Dec, 31	Jan, 1	Jan, 15
			Dec, 22	Dec, 26	Dec, 31	Jan, 1	Jan, 15
			Dec, 26	Dec, 28	Jan, 2	Jan, 3	Jan, 15

### Culturing of embryonic fibroblast-like cells

We used the method of enzymatic disaggregation to isolate cells: the embryos were placed in a Petri dish (diameter 6 cm) with 2 ml of 0.25% trypsin (PanEco, Russia), crushed into pieces 1–0.5 mm in size. Then 3 ml of trypsin was added and incubated in a thermostat at 37° C for 15–30 min, pipetted every 5 min. Then it was centrifuged for 7 min at 100 g, the pellet was resuspended in a medium with 10% fetal bovine serum^37^. The cells were cultured in DMEM/F12 medium with l-glutamine (PanEco, Russia) 10% FBS (Biosera France) and 0.004% gentamicin (Borisov Medicines Plant, Belarus) at 37 °C in a humid atmosphere containing 5% CO_2_^37^.

1 × 10^6^, 1.5 × 10^6^ and 2 × 10^6^ of cells in a volume of 10 ml of complete grows medium were cultured in 25 cm^2^ cultural flask (T-25, Corning, USA) during 4 h then medium was changed to new one. In 3 days, the cells from the flask, in which 2 × 10^6^ cells were planted, were transplanted onto a similar flask for further cultivation in an amount of 0.75 × 10^6^. After 4 days, a similar procedure was performed for a flask into which 1.5 × 10^6^ cells were added, and after 5 days, with a flask where the minimum number of cells was added (1 × 10^6^).

**Figure 9:**
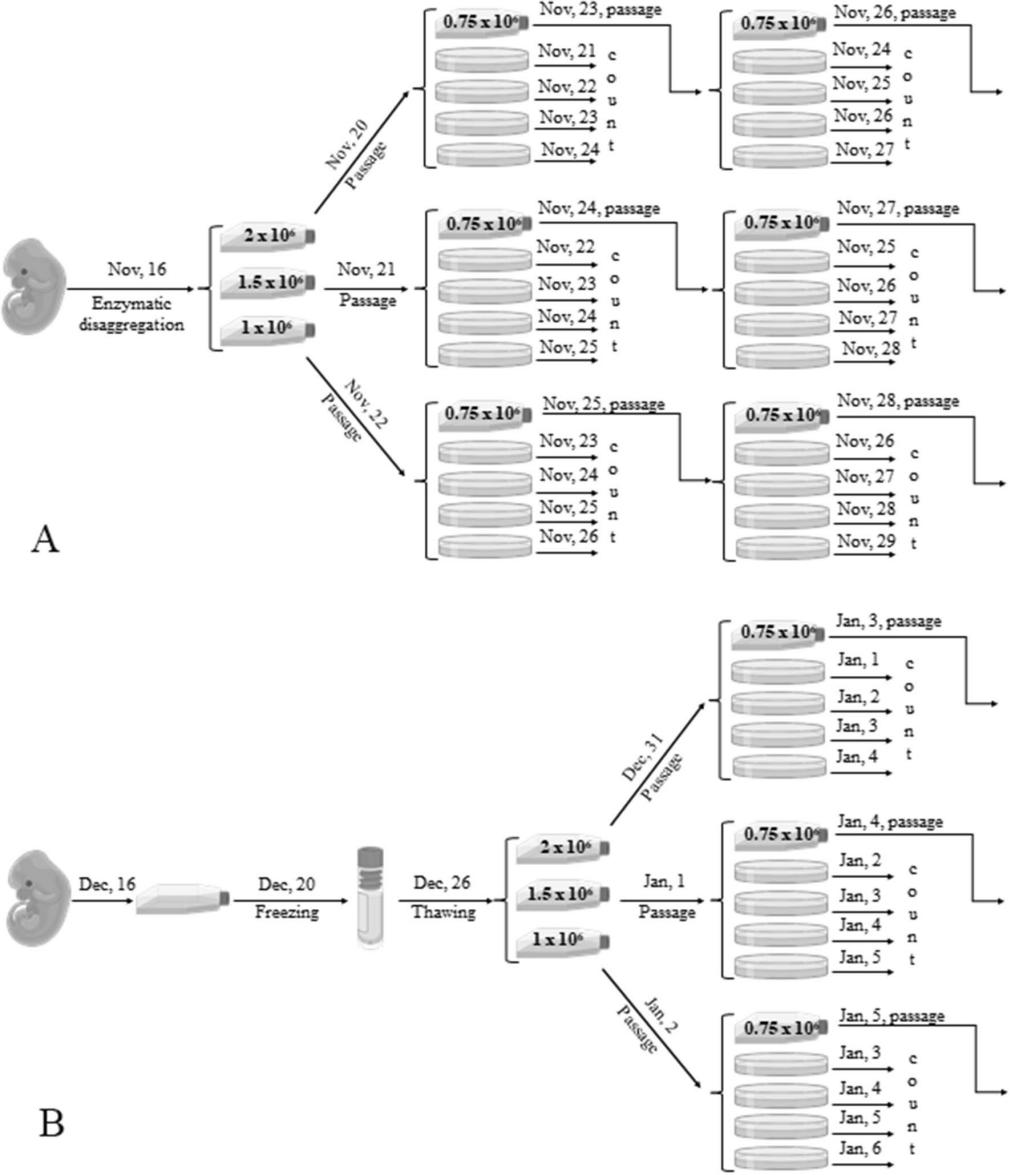
Scheme of the experiments to identify an infradian rhythm of the embryonic fibroblast-like cell culture proliferative activity in the logarithmic growth phase (**A**) and after freezing and thawing of the culture of embryonic fibroblast-like cells (**B**).

After subculturing, cells in the amount of 300,000 were plated on 12 Petri dishes with a diameter of 6 cm in a volume of 5 ml of medium. To standardize the experiment and the constant presence of cells in the logarithmic growth phase, the cells were seeded in Petri dishes daily during the study period. The experimental scheme is shown in Table 3 and Fig. 9A.

**Table 3:**
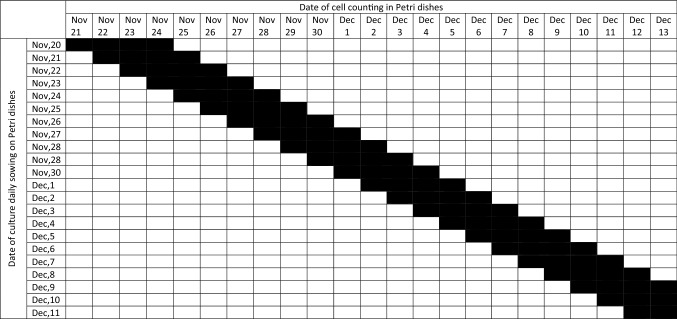
Date of cell counting in embryonic cells culture.

*Black block—is a date of cell counting of embryonic fibroblast-like cell culture.

Each subsequent day, for the purpose of counting, the cells were removed from 2 Petri dishes by trypsinization: 2 ml of 0.02% chymotrypsin diluted in Versene solution was added and incubated for 4 min at 37 °C. Cells were counted with using an automatic cell counter TC-20 (Bio-Rad Laboratories, USA).

### Freezing and thawing of embryonic fibroblast-like cells

To change the phase of the infradian rhythm of the proliferative activity of embryonic fibroblast-like cells, they were exposed to short-term freezing. Culture samples from the embryos of one female were frozen on December 20, 22 and 26. Procedure of freezing included the next steps: 2–3 × 10^6^ cells were added to 0.5 ml of cryopreservation medium (90% FBS and 10% DMSO), the cell suspension was poured into ampoules for freezing. To ensure a cooling rate of 1 °C/min, the ampoules were placed on cotton wool in a polystyrene foam box with walls 15 mm thick and put them into a − 70 °C fridge. After 2 h, when the ampoules reached − 70 °C, they were immersed in a liquid nitrogen freezer.

In 2, 4 and 6 days after freezing, two cells cultures were defrosted in a thermostat at 37 °C, respectively, on December 26 and 29, 2019. After defrosting, a small amount of growth medium was slowly added to the ampoule, then the contents of the ampoule were transferred to a centrifuge tube and the medium was slowly added to the end volume of 10 ml. It was centrifuged for 3 min at 100 g. The cells were resuspended in 10 ml fresh growth medium and then transferred to 25 cm^2^ culture flasks (T-25, Corning, USA) in amount of 1 × 10^6^, 1.5 × 10^6^ and 2 × 10^6^. In 3 days, the cells from the flask, in which 2 × 10^6^ cells were planted, were transplanted onto a similar flask for further cultivation in an amount of 0.75 × 10^6^. After 4 days, a similar procedure was performed for a flask into which 1.5 × 10^6^ cells were added, and after 5 days, with a flask where the minimum number of cells was added (1 × 10^6^).

As embryonic cells are more sensitive to freezing and they show low proliferative activity in the first passage immediately after thawing, therefore at first they were cultured in a flask for 5 days. And only after that they were plated on Petri dishes for the study of proliferative activity. The experimental and cell counting scheme did not differ from that presented above for all other cultures (Table 3, Fig. 9B).

### Culturing of L929 cells

The L929 culture of transformed mouse fibroblasts was obtained from the collection of vertebrate cell cultures of the Institute of Cytology, Russian Academy of Sciences (Russia, St. Petersburg). The L929 cells were cultured in growth medium DMEM/F12 with l-glutamine (Capricorn Scientific, Germany), 10% FBS (Biosera France), 50 U/ml penicillin and 50 μg/ml streptomycin (PanEco, Russia).

To obtain a series of identical samples, cells after several passages were simultaneously frozen. DMSO (dimethyl sulfoxide) was used as a cryoprotectant for freezing the L929 culture. The cell suspension of L929 was frozen in 10% DMSO and 90% of cattle serum, for this, 2.5 × 10^6^ cells were diluted in 1 ml of freezing medium and placed in cryotubes. To ensure cooling of cryoprobes at a rate of 1 °C/min, the cryotubes were placed on cotton wool in a polystyrene foam box with 15 mm wall thickness, then put into a low-temperature freezer at − 70 °C.

The culture was thawed by placing it in a thermostat for 5 min at 37 °C, then centrifuged at 200*g* for 4 min, resuspended in a growth medium and centrifuged again. After centrifugation, it was diluted in 2 ml of growth medium. Viable cells were counted on an automatic cell counter TC-20 (Bio-Rad Laboratories, USA). Viable cells of one cryoprobe with 150 × 10^3^ cells in 3 ml of growth medium were plated on Petri dishes 35 mm in diameter.

### Freezing and thawing of L929 cells

**Figure 10:**
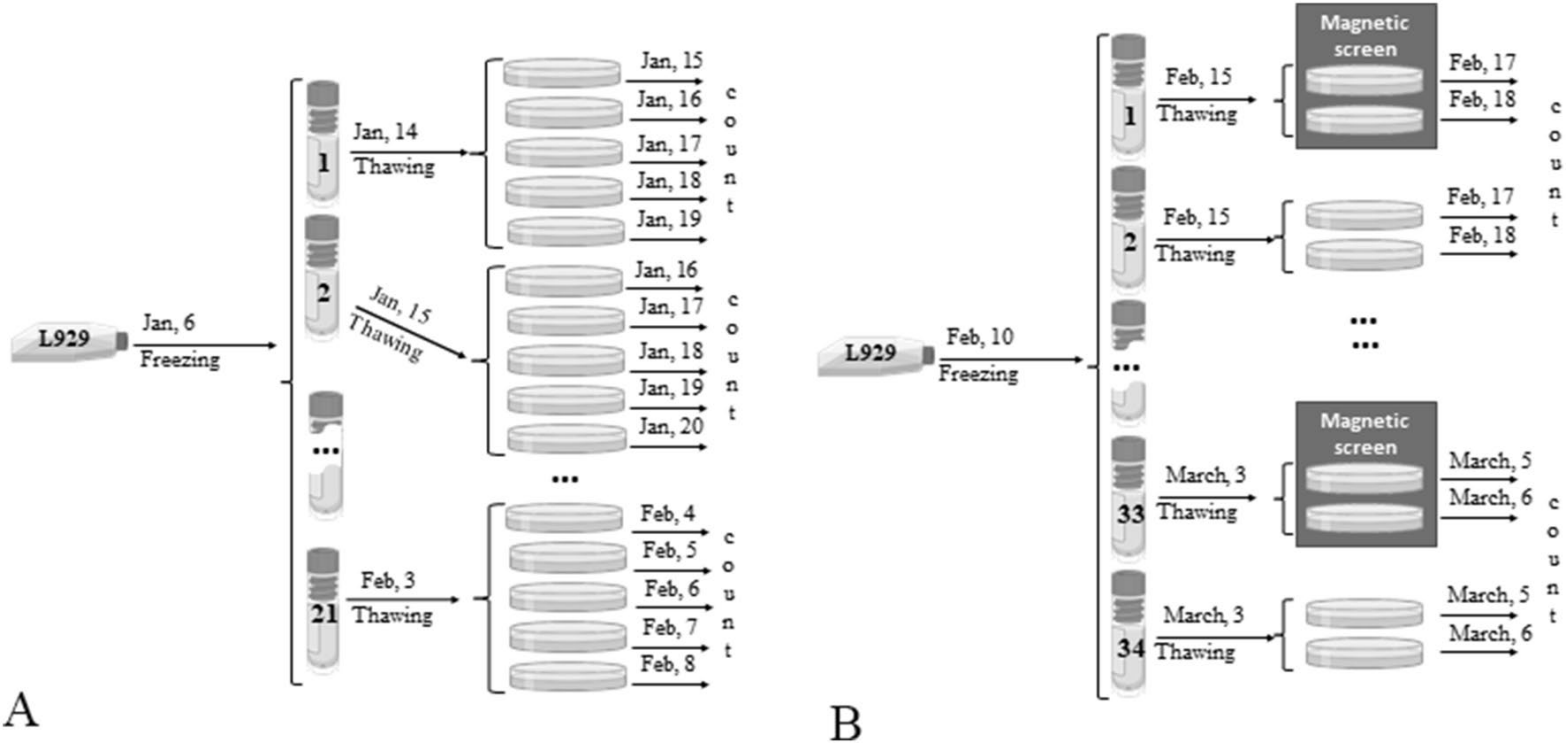
Scheme of the experiment to identify the infradian rhythm of the proliferative activity of the L929 cell culture after freezing and daily thawing (**A**) and in the condition of the permalloy magnetic screen and without it (**B**).

We carried out two series of experiments with freezing/thawing of the L929 culture. To assess the proliferative activity of cells in the logarithmic growth phase, every day from January 14 to February 3 and from February 15 to March 3, 2021 we thawed one cryoprobe (Table 4). In the first series, the indicators of the increase in the cells number after the 2nd, 3rd, 4th and 5th days of cultivation were analyzed, while the cells were counted daily from one of the Petri dishes (Tables 5 and 6). In the second series, the indicators were obtained only after the 2nd and 3rd days of cultivation, three Petri dishes from each cryoprobe were analyzed daily (Tables 5 and 6, Fig. 10A).

**Table 4:** Calendar date of experimental manipulations with L929 cells.

№ series	Freezing date	Defrost date of the first cryosample	Defrost date of the last cryosample	Number of repetitions	Number of days after seeding, when the determination of the number of cells in the Petri dish was carried out	The duration of the obtained data series of the daily increase in the number of cells
1	Jan, 6	Jan, 14	Feb, 3	1	1, 2, 3, 4, 5,	22 days
2	Jan, 10	Jan, 15	March, 3	3	2, 3	17 days

**Table 5:**
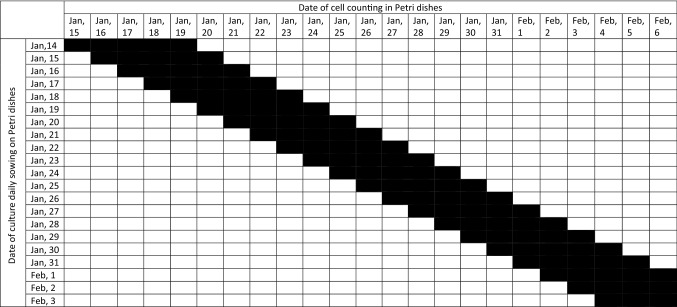
Date of cell counting in L929 cells culture from January, 15 to February, 6, 2021.

*Black block—is a date of cell counting of L929 culture.

**Table 6:**
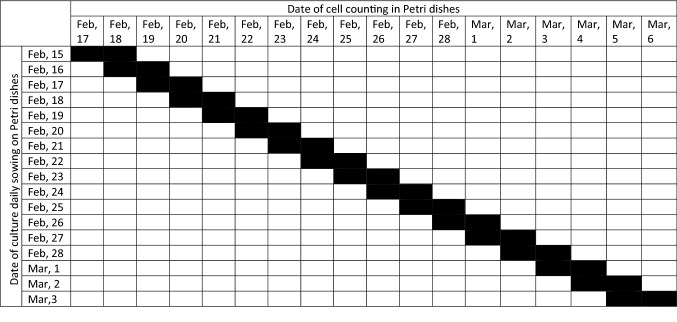
Date of cell counting in L929 cells culture from February, 17 to March, 6, 2021.

*Black block—is a date of cell counting of L929 culture.

### Motor activity of mice

To compare the phases of the 4-day infradian rhythm in the fibroblast-like cell culture and in the organism we determined the rhythm of motor activity of mice. We used 7 male C57Bl/6 mice for that. The motor activity of the animals was determined with intraperitoneally implanted DTN4-28/TL4-28 sensors (EMBI RESERCH, Novosibirsk). Implantation of sensors into the abdominal cavity had been carried out under anesthesia by Zoletil (Virbac Sante Animale, France) at the dose of 5–7 mg/kg. Measurements were carried out at a frequency of 1 time per minute. The total daily activity was determined as the sum of the minute readings of the accelerometer built into the sensor.

### The rhythmicity of the proliferative activity of the L929 mice cells in conditions of the electromagnetic shielding

To assess the contribution of the variability of the Earth's magnetic field to the 4-day infradian biological rhythm of the L929 cell proliferative activity, we studied the increase in the number of cells in a L929 culture located in a magnetic permalloy screen that 35-fold attenuates the total geomagnetic field. Since it has been shown that a decrease in the magnetic field by more than 100 times leads to pronounced negative effects in the functioning of the body and cells—a decrease in the activity of some enzymes and cell proliferation, the use of a screen that reduces fluctuations in the Earth's magnetic field by 100–1000 times can hide the studied infradian rhythm^38–42^.

The magnetic screen was made in the form of two coaxial cylinders that fit one into the other. The wall of each of the cylinders consisted of 3 sheets of permalloy 0.5 mm thick each. The gap between the walls of the cylinders was 20 mm. For air circulation, holes with a diameter of 15 mm were drilled in each of the cylinders, while these holes in the outer and inner cylinders were not located opposite each other. The coefficient of attenuation of the Earth's magnetic field by this screen was determined using an FL3-100 three-component sensor (Stefan Mayer Instruments GmbH & Co. KG, Germany).

The scheme of the experiment is shown in the Fig. 10B. On February 10, identical aliquots of the L929 culture were frozen. From February 15 to March 3, two aliquots were thawed daily, one of which was seeded on six Petri dishes and placed in a magnetic screen, and the other aliquote (control group) was seeded on 6 Petri dishes too but incubated outside the magnetic screen. The number of cells was determined in 3 repetitions (the average was calculated for three identical Petri dishes), two and three days after incubation.

### Statistical analyzes

Statistical analyzes was performed using the Statistica 7.0 software package. The obtained data were expressed as the median and interquartile range Me (Q1–Q3). To identify the periods of infradian rhythms, the autocorrelation coefficient was calculated between the original series and the series shifted by 1, 2, 3, and 4 days. In order to identify the synchronicity of changes in the dynamics of the studied parameters, the Spearman correlation coefficient was calculated. Differences were considered statistically significant at p < 0.05.
